# Alleviation of Lead Stress on Sage Plant by 5-Aminolevulinic Acid (ALA)

**DOI:** 10.3390/plants10091969

**Published:** 2021-09-21

**Authors:** Hamed M. El-Shora, Gehan F. Massoud, Ghada A. El-Sherbeny, Salma Saleh Alrdahe, Doaa B. Darwish

**Affiliations:** 1 Botany Department, Faculty of Science, Mansoura University, Mansoura 35511, Egypt; drghadaelsherbeny@yahoo.com (G.A.E.-S.); ddarwish@ut.edu.sa (D.B.D.); 2 Agricultural Research Centre, Medicinal and Aromatic Plants Research Department, Horticulture Research Institute, Cairo 12619, Egypt; gehan.fawzy75@gmail.com; 3 Department of Biology, Faculty of Science, University of Tabuk, Tabuk 71491, Saudi Arabia; salrdahe@ut.edu.sa

**Keywords:** *Salvia officinalis*, lead, 5-aminolevulinic acid, germination, malondialdehyde, antioxidant enzymes

## Abstract

Oxidative stress is imparted by a varying range of environmental factors involving heavy metal stress. Thus, the mechanisms of antioxidant resistance may advance a policy to improve metal tolerance. Lead as a toxic heavy metal negatively affects the metabolic activities and growth of medicinal and aromatic plants. This investigation aimed to assess the function of 5-aminolevulinic acid (ALA) in the alleviation of Pb stress in sage plants (*Salvia officinalis* L.) grown either hydroponically or in pots. Various concentrations of Pb (0, 100, 200, and 400 µM) and different concentrations of ALA (0, 10, and 20 mg L^−1^) were tested. This investigation showed that Pb altered the physiological parameters. Pb stress differentially reduced germination percentage and protein content compared to control plants. However, lead stress promoted malondialdehyde (MDA) and H_2_O_2_ contents in the treated plants. Also, lead stress enhanced the anti-oxidative enzyme activities; ascorbate peroxidase superoxide, dismutase, glutathione peroxidase, and glutathione reductase in *Salvia* plants. ALA application enhanced the germination percentage and protein content compared to their corresponding controls. Whereas, under ALA application MDA and H_2_O_2_ contents, as well as the activities of SOD, APX, GPX, and GR, were lowered. These findings suggest that ALA at the 20 mgL^−1^ level protects the *Salvia* plant from Pb stress. Therefore, the results recommend ALA application to alleviate Pb stress.

## 1. Introduction

Plants are immobile in their nature, and they are subjected to various abiotic and biotic stresses. Therefore, they develop different strategies to cope with stress conditions and mitigate the toxic effect. Heavy metals are toxic pollutants that are tough to eliminate and small quantities can cause an unlimited threat to the environment [1]. Heavy metals produce oxidative stress and desiccation in plants, which result in the reduction of the yield and corrosion in the crop quality [2,3,4]. Photosynthesis and PSII are sensitive to heavy metals and particularly lead stress. The high concentration of lead can cause a number of poisonous symptoms in plants including negative effects on photosynthesis. Lead effects have been illustrated for both donor and acceptor sites of photosynthesis-2 (PS II), the cytochrome b/f complex, and photosynthesis-1 (PSI). It is commonly known that the PSI electron transport process is less sensitive to inhibition by lead than photosynthesis-2 (PSII) [5,6].

Lead is the most abundant hazardous metal in the environment, where it is not an essential nutrient for plants, and it is regarded as a toxic heavy metal due to its widespread distribution and its extensive environmental and human health problems [7]. Pb(NO_3_)_2_ is a causal source of pollution in ecosystems [8]. Its major sources are chimneys of factories, pesticides, fertilizers, and exhausts of automobiles [9]. Lead accumulation is recognized to cause highly deleterious effects on the growth and yield of plants [10], where it was reported to induce the production of reactive oxygen species (ROS) and cause oxidative damage to nucleic acids, proteins, and lipids [11,12] also, Pb-induced ROS scavenging enzymes in plants [13,14]. 

The plant growth hormones are often used to relieve abiotic stress in plants. ALA is considered one of the most vigorous plant growth regulators and the first compound in the pathway of porphyrin synthesis, which leads to the speeding up of the synthesis of chlorophyll in plants [15]. It has been found that ALA performs a crucial part in sustaining optimum plant growth and it improves crop yields [16]. ALA is known to stimulate plant development and reactions to several stresses [17,18]. It was found that ALA induces stress tolerance [19,20] and revival of growth under herbicide stress [21]. 

*Salvia officinalis* L. (sage) belongs to the Family Lamiaceae. This species is native to the Middle East and Mediterranean areas and recently it has been naturalized throughout the world particularly in Europe and North America [22,23]. It is used in folk medicine as herbal tea and antiseptic, antihydrotic, and anti-inflammatory medicine where it has curative properties for ulcers, gout, dizziness, rheumatism, hyperglycemia, and seizures [24,25]. Also, it is used as a food additive as dried leaves [26]. For all these fantastic medicinal and cookery potentialities, *S. officinalis* is cultivated and many Mediterranean countries, where sage grows, have substantial gains from its production and export [27]. Lead is counted as a strong environmental pollutant. Different ecological, evolutionary, and environmental processes in the microsphere are disordered because of lead toxicity to the microbial community. Based on this vital evaluation, the effects of increasing doses (control, 100, 200, and 400 µM) of lead nitrate as heavy metal on seed germination and the antioxidant capacity of sag seedlings were investigated. Also, the present work aimed to investigate the influence of ALA on the mitigation of lead toxicity in sage plants. 

## 2. Materials and Methods

### 2.1. Plant Material

Laboratory and greenhouse experiments using pots were designed. The seeds of *Salvia officinalis* L. were obtained from the Medicinal and Aromatic Plants, Department of Horticulture Research Institute, Agriculture Research Center, Egypt and selected for apparent uniformity in size and shape.

### 2.2. Germination of Salvia officinalis Seeds

The seeds of *Salvia* were germinated as described by the authors of [28]. Healthy seeds were surface treated with a 0.5% (*v/v*) aqueous solution of HgCl_2_ for 1–2 min followed by repeated washing with distilled water. The seeds were divided into three groups and raised on moist filter paper in Petri dishes. The first group was soaked in distilled water as a control. The second group was primed in 10 mgL^−1^ ALA solution for 12 h. The third group was primed in 20 mgL^−1^ ALA solution for the same period of time. Each group of primed seeds was divided into three sub-groups to be watered for 7 days with Hoagland solution [29] containing 100, 200, and 400 µM of Pb(NO_3_)_2._ For the pot experiment, the seeds were germinated in small plastic pots with sandy soil. Seeds were considered to be germinated on the first appearance of the radicle. Germination % = Number of germinated seeds/Total number of seeds [30].

### 2.3. Treatment of Salvia officinalis Seedlings with Pb(NO_3_)_2_

The non-primed seeds (control) and the ones primed in 10 or 20 mgL^−1^ ALA were left to germinate for days in sterile Petri dishes or in plastic pots with sandy soil of 70% relative activity. All seeds were watered with Hoagland solution free from Pb(NO_3_)_2_ for 7 days. Each group of primed seeds in 10 or 20 mgL^−1^ ALA was then divided into three sub-groups. The first sub-group was transported to a Hoagland solution with 100 µM Pb(NO_3_)_2_. The second sub-group was transferred to a Hoagland solution with 200 Pb(NO_3_)_2_ and the third sub-group was shifted to a Hoagland solution with 400 µM Pb(NO_3_)_2_. All sub-groups of seedlings were left to grow for 14 days in a growth chamber under controlled conditions (12:12 day/night, 25/30 °C±2, and photon flux density of 95 µmol m^2^s^−1^. The hydroponic system was continuously aerated using an air pump and the solution was renewed each 48 h.

### 2.4. Preparation of Leaf Extract of Salvia officinalis

Samples of plant leaves were weighed and homogenized in a pestle and motor with extraction buffer (pH 7.0) then centrifuged for 10 min at 10,000× *g* rpm. The obtained supernatant represented the plant leaf extract.

### 2.5. Determination of Total Soluble Protein (TSP) Content

The TSP content of leaves of *Salvia officinalis* L. was determined by the method in Ref. [31]. A sample of 30 μL of leaf extract was added to a tube and the volume was made up to 100 μL with 0.15 M NaCl. Then, 1 mL of Bradford’s reagent was added and mixed well and the absorbance at 595 nm was determined. The protein concentration in the sample using the calibration curve of bovine serum albumin (BSA) as a standard was measured.

### 2.6. Determination of MDA Content

Lipid peroxidation was investigated by the estimation of the malondialdehyde (MDA) content by the method in Ref. [32]. Samples (200 mg) of the frozen leaves were ground in 3 mL of 0.2% (*w/v*) TCA and then centrifuged. A sample (0.2 mL) of the resulting supernatant was combined with 2 mL of 25% (*w/v*) TCA including 0.4% thiobarbituric acid (TBBA) and incubated for 35 min at 95 °C. The mixture was cooled in an ice bath and then centrifuged at 10,000× *g* rpm for 15 min. The absorbance was recorded at 532 nm.

### 2.7. Determination of Hydrogen Peroxide Content

Determination of H_2_O_2_ content was carried out following Ref. [33]. A sample (0.5 g) of plant leaves was homogenized with liquid nitrogen then suspended in chilled 5 mL of 0.1% (*w/v*) TCA. The homogenate was then centrifuged at 13,000× *g* rpm for 20 min. A sample of 0.5 mL of supernatant was mixed with 0.5 mL of 10 mM potassium phosphate buffer (pH 7.0) and 1 mL of M KI. The reaction was left for 1 h in darkness followed by measuring the absorbance at 390 nm. H_2_O_2_ concentration was determined by using a standard curve.

### 2.8. Determination of Antioxidant Enzyme Activity

#### 2.8.1. Preparation of Enzymes Extract

Enzyme extract was prepared Ref. [34] where one gm of fresh leaves from control and treated plants was homogenized with 4 mL of 150 mM phosphate buffer (pH 7.0) containing 1 mM polyvinylpyrrolidone (1%) and 1 mM EDTA followed by centrifuging at 15,000× *g* rpm at 4 °C for 25 min. The resulting supernatant represented the crude extract for the enzymes assay.

#### 2.8.2. Assay of Enzymes

##### Assay of Ascorbate Peroxidase (APX, EC: 1.11.1.11)

The activity of ascorbate peroxidase (APX) was determined by the method in Ref. [35]. It is a spectrophotometric method where the rate of decrease in absorbance of ascorbate during its oxidation is measured at 290 nm wavelength. Half gram of plant leaves was mixed with 10 mL of phosphate buffer (50 mM pH 7.5) comprising 0.3 mM EDTA and homogenized in a pestle and mortar followed by centrifuging for 25 min at 10,000× *g* rpm at 4 °C. 

The supernatant was transported to another tube and used as enzyme extract. The enzyme was assayed in 1 mL cuvette containing 350 µM ascorbate, 0.5 mM EDTA, 2 mM H_2_O_2_, 100 mM potassium phosphate buffer (pH 7.0), and 100 µL enzyme extract. The rate of reduction in absorbance at 290 nm in the course of ascorbate oxidation was read. 

##### Assay of Glutathione Peroxidase (GPX, EC: 1.11.1.9)

Glutathione peroxidase (GPX) activity was estimated by [36]. Plant extract (50 µL), 3 mL of extraction buffer (pH 7.5), 0.3 mL of sodium azide and 0.2 mL EDTA were mixed. Glutathione (2 mL) and 0.2 mL of H_2_O*_2_* were included in the mixture and incubated at 37 °C for 10 min followed by termination of the reaction after 15 min by 0.5 mL of 10% TCA. The samples were then centrifuged. The supernatant was assayed for glutathione as mentioned previously. 

##### Assay of Superoxide Dismutase (SOD, EC: 1.15.1.1)

SOD activity was determined following Ref. [37]. The assay is based on its capacity to hinder the photochemical reduction of the nitro blue tetrazolium (NBT). The assay mixture (3 mL) contained 100 mM phosphate buffer (pH 7.8), 20 mM methionine, 520 µM NBT, 10 µM EDTA, 30 µM of riboflavin, a sample (50 µL) of the enzyme extract, and 300 µL the reaction was allowed to stand 20 min under 4000 lx light. The absorbance was taken at 560 nm.

##### Assay of Glutathione Reductase (GR, EC: 1.6.4.2)

GR assay was carried out according to Ref. [38] by measuring the reduction in absorbance at 334 nm due to NADPH oxidation. The assay mixture of 3.0 mL contained 1 mM oxidized glutathione (GSSG), 2 mM EDTA, 150 mM extraction buffer (pH 7.5), 0.4 mM NADPH, and 100 µL enzyme preparation at 30 °C.

### 2.9. Statistical Analysis

The experimental design was randomized completely. All values represent the mean of four replicates. Data were exposed to ANOVA and examined by Duncan’s multiple range tests at 0.05 probability level using the COSTAT 6.3 program. Values represent the mean ± SD (*n* = 3). Means monitored by the similar letter did not significantly vary at *p* ≤ 0.05 fitting to Duncan’s multiple range tests.

## 3. Results

**Figure 1:**
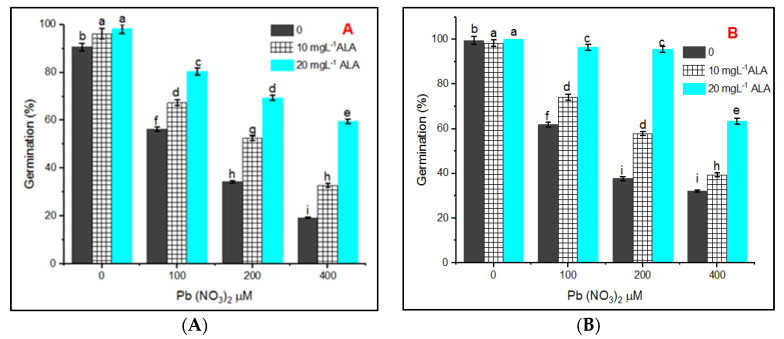
Effect of various concentrations of Pb(NO_3_)_2_ on seed germination of *Salvia officinalis* and stress alleviation by ALA in hydroponic (**A**) and pot experiments (**B**).

The data in Figure 1A,B indicate that the germination percentage of *Salvia officinalis* seeds in hydroponic and in pot experiments were decreased gradually with an increase in the concentration of Pb(NO_3_)_2_ (100, 200, and 400 µM). The germination percentage of *S. officinalis* seeds in Petri dishes treated with 400 µM solution was 19.33% and 23.33% at both Hoagland’s nutrient solutions and pot experiments, respectively.

Priming seeds in 10 or 20 mg L^−1^ of ALA, the application of ALA increased the percentage of germination of *S. officinalis* seeds under all the tested concentrations of Pb (100, 200, and 400 µM) in both experiments. ALA at 20 mgL^−1^ was most effective in the alleviation of lead stress compared to 10 mgL^−1^ of ALA. 

**Figure 2:**
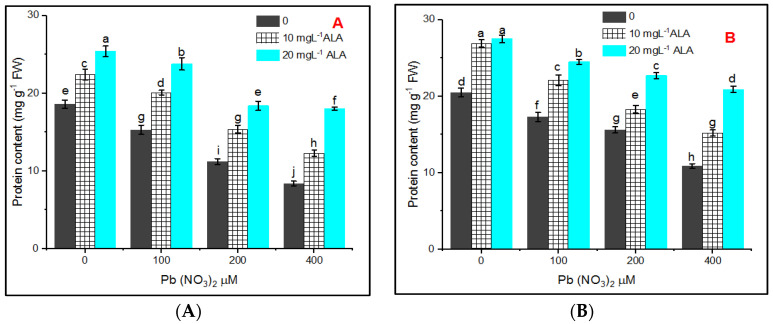
Effect of various concentrations of Pb(NO_3_)_2_ on total soluble protein content in leaves of *Salvia officinalis* and stress alleviation by ALA in hydroponic (**A**) and pot experiment (**B**).

The results represented in Figure 2A,B showed that the total soluble protein content in *S. officinalis* leaves grown in hydroponic and in pot experiments was reduced under the various tested concentrations of Pb and the reduction was concentration-dependent. Alleviation of Pb stress in *S. officinalis* plants in hydroponic or pot experiments was dependent on ALA concentration.

**Figure 3:**
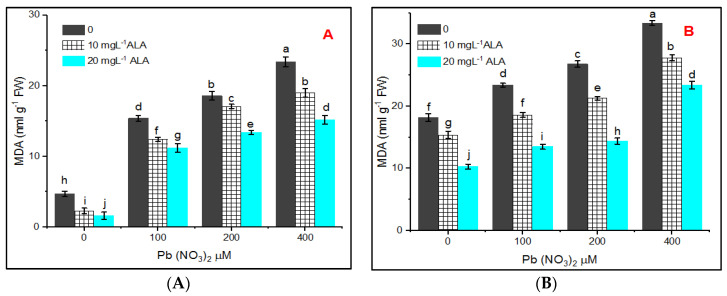
Effect of various concentrations of Pb(NO_3_)_2_ on MDA content in leaves of *Salvia officinalis* and stress alleviation by ALA in hydroponic (**A**) and pot experiment (**B**).

Lipid peroxidation (MDA) as a marker of stress was determined to investigate the oxidative stress in *S. officinalis* under Pb treatment. The illustrated data in Figure 3A,B indicated a low level of MDA in control plants, however, in Pb-treated plants, MDA increased gradually depending on the concentrations of Pb. The highest value was recorded under treatment with 400 µM Pb concentration. Application of ALA resulted in reducing H_2_O_2_ contents by 18.57% and 34.96% in hydroponic as well as 16.84% and 30% in pot experiment at 10 and 20 mg L^−1^ level of ALA.

**Figure 4:**
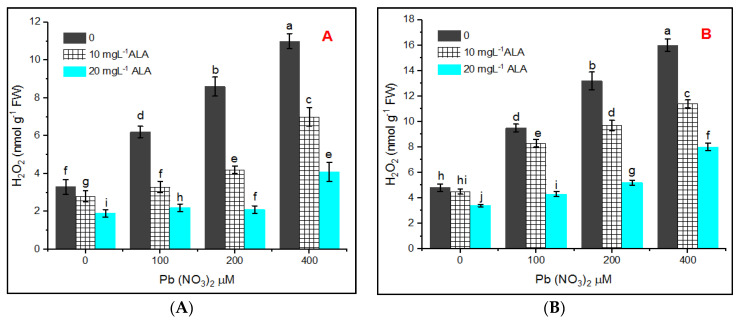
Effect of various concentrations of Pb(NO_3_)_2_ on H_2_O_2_ content in leaves of *Salvia officinalis* and stress alleviation by ALA in hydroponic (**A**) and pot experiment (**B**).

Production of both hydrogen peroxide (H_2_O_2_ and MDA) was increased apparently in response to Pb stress, and the increase was significantly counteracted by priming in ALA. The obtained results indicated the presence of low concentrations of H_2_O_2_ in plants whose seeds were treated with water, 10 and 20 mgL^−1^ of ALA (Figure 4A,B).

However, H_2_O_2_ increased gradually under Pb treatments in a concentration-dependent manner. The highest contents of H_2_O_2_ in leaves of treated plants were 11.0 and 16.00 nmol g^−1^ FW at 400 µM Pb in hydroponic and pot experiments, respectively. Priming of *S. officinalis* seeds in 10 or 20 mgL^−1^ of ALA resulted in partial alleviation of Pb stress.

**Figure 5:**
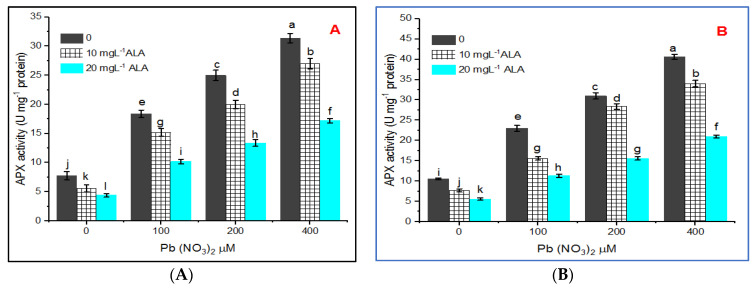
Effect of various concentrations of Pb(NO_3_)_2_ on APX activity of *Salvia officinalis* leaves and stress alleviation by ALA in hydroponic (**A**) and pot experiment (**B**).

The alleviation of lead toxicity was represented by 29.07% and 50.09% in the presence of 10 or 20 mg L^−1^ of ALA, respectively. Pb stress potentially disturbed the enzymatic activities of *S. officinalis* plants. This experiment indicated an appreciable activity of ascorbate peroxidase (APX) in the control plants (Figure 5A,B).

However, APX increased progressively with increasing Pb concentrations in the leaves of *S. officinalis* plants whose seeds were primed or not primed in ALA. The highest activities were 31.43 and 40.57 units mg^−1^ protein in hydroponic and pot experiments, respectively. In the treatment with 400 µM of Pb, the increase was about five times that of the control. Priming seeds in ALA decreased APX activity and the ameliorative role of ALA was more apparent with 10 than 20 mg L^−1^ in hydroponic and pot experiments.

**Figure 6:**
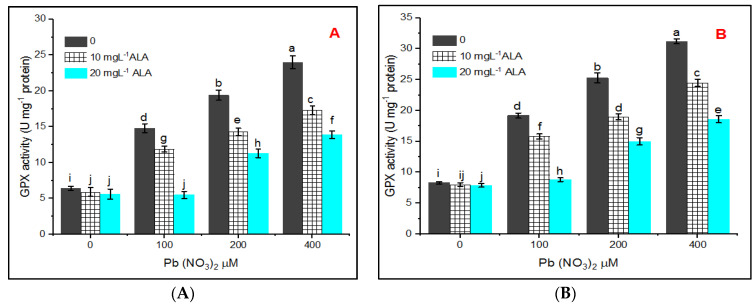
Effect of various concentrations of Pb(NO_3_)_2_ on GPX activity of *Salvia officinalis* leaves and stress alleviation by ALA in hydroponic (**A**) and pot experiment (**B**).

Regarding the activity of glutathione peroxidase (GPX), it takes place in the same manner as the other antioxidant enzymes. As indicated in Figure 6A,B, GPX increased under treatments with Pb at various concentrations. Priming seeds in ALA lowered the activity of the GPX enzyme and 20 mg L^−1^ of ALA was more effective than 10 mg L^−1^. This was recorded for plants grown in hydroponic and pot experiments.

**Figure 7:**
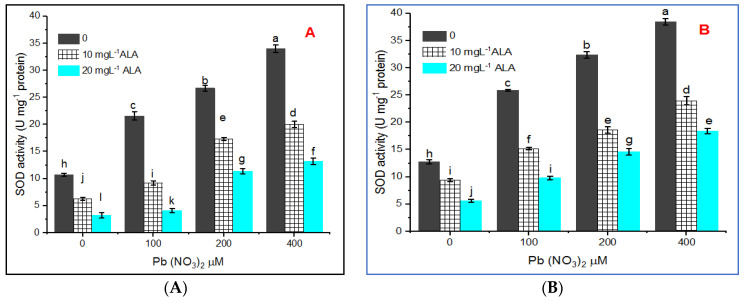
Effect of various concentrations of Pb(NO_3_)_2_ on SOD activity of *Salvia officinalis* leaves and stress alleviation by ALA in hydroponic (**A**) and pot experiment (**B**).

Concerning superoxide dismutase (SOD), the results illustrated in Figure 7A,B showed that low activity was observed in control plants and enhanced under Pb treatment with various concentrations. However, SOD increased continuously with increasing the concentration of Pb. The highest activities of SOD were 34.0 and 38.37 units mg^−1^ protein recorded at 400 µM Pb in hydroponic and pot experiments, respectively. 

ALA either at 10 or 20 mg L^−1^ ameliorated partially the induced stress by Pb treatments on *Salvia* plants and this was apparent by the lowered activity of SOD under the treatments with various concentrations of Pb. The enzyme activities were decreased by 41.09% and 61.26% under Pb 400 µM treatment in hydroponic experiments with 10 and 20 mg L^−1^ ALA. Also, SOD activity was lowered by 37.45 and 52.12% in pot experiments under Pb 400 µM at ALA 10 and 20 mg L^−1^ level, respectively.

**Figure 8:**
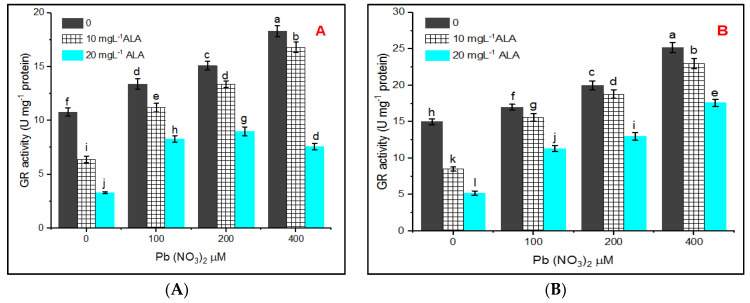
Effect of various concentrations of Pb(NO_3_)_2_ on GR activity of *Salvia officinalis* leaves and stress alleviation by ALA in hydroponic (**A**) and pot experiments (**B**).

The results indicated in Figure 8A,B showed that GR activity increased under all Pb treatments. However, ALA treatment lowered the GR activity, lowering in the rate of activity at 20 mg L^−1^ ALA was higher than that recorded at 10 mg L^−1^ of ALA.

## 4. Discussion

The results obtained in the present study showed that the treatment of *Salvia officinalis* with Pb (0, 100, 200, 400 µM) showed a considerable alteration in the measured physiological parameters in both hydroponic and pot experiments. Thus, there is a reduction in the percentage of seed germination of S. *officinalis*, which may be attributed to the fact that Pb treatment reduced the rate of cell metabolic processes such as cell division, nucleic acid synthesis [39] and Pb impaired seedling development, as well as chlorophyll production [40]. In addition, increasing levels of Pb in soil inhibited the germination of seeds and affected the plant metabolism [41]. 

On the other hand, plant growth was improved under the combined treatment of ALA (10 and 20 mg L^−1^) and Pb stress. This increase in seed germination with ALA might be owing to the fact that ALA has an enhancing effect in regulating various metabolic processes, due to this behavior, the growth and yield of most plants under abiotic stresses have been improved [42]. It has been reported that ALA improved plant growth by alleviating cadmium [43] and lead effects in oilseed rape (*Brassica napus*).

The present results regarding total soluble protein (TSP) revealed that there is a decline in TSP content with increasing Pb concentration in untreated sage plants with ALA. Tamás et al. [44] and El-Shora et al. [45] reported that Pb treatment reduced protein content, which may be attributed to oxidative modifications by ROS [46] produced under Pb stress [47,48,49]. Also, priming seeds in ALA possibly increases the defense system through induction of antioxidant synthesis [50]. 

The data indicated that S. *officinalis* treated with both concentrations of ALA before Pb treatment showed an increase in MDA content comparing to the control. These results are in harmony with the fact that plants grown under stressed conditions create free radicals leading to lipid peroxidation of membranes and increasing MDA content [51,52]. Lipid peroxidation is a biochemical marker for the free radical-mediated injury with increasing lead concentration in *Talinum triangulare* leaves [53,54]. MDA is responsible for cell membrane damage, and it is one of the final products of peroxidation of unsaturated fatty acids in phospholipids [55]. In lipid peroxidation, the free radicals take away electrons from membrane lipid leading to cell membrane damage [56]. 

The level of ALA in plants originated from primed seeds in water or ALA expressed lower content of H_2_O_2_ compared to those treated with various concentrations of Pb. The increase in H_2_O_2_ content as one of ROS in *Salvia* leaves under Pb treatments, whether in hydroponic or pot experiments, reflected the oxidative stress stimulated by Pb and these results agreed with those reported by the authors of [57] and might be due to the destabilization of membranes by increasing Pb levels. It has been reported that ROS were produced under stress conditions and are eliminated by antioxidant systems [58]. Pb toxicity enhances the ROS production in mitochondria, chloroplasts, and peroxisomes [59]. Rasheed et al. [60] reported that application of ALA diminishes the levels of both MDA and H_2_O_2_ and consequently reduces the oxidative damage in sunflower subjected to water stress.

Higher plants have an antioxidant system that diminishes oxidative stress. This system involves antioxidant enzymes that support plants with adaptation and survival under various stresses and oxidative damage [61,62]. The results reveal a higher level of antioxidant activities in plants whose seeds were primed in ALA at 10 and 20 mg L^−1^ than those of the control plants. It seems likely that ALA enhanced the activities of the various enzymes of ROS hunting. In plants grown from seeds primed in ALA and treated with Pb, the activities of the four estimated antioxidant enzymes (APX, GPX, SOD, and GR) improved continuously but with slower rates compared to the plants grown from seeds primed in water possibly because Pb concentrations seem likely to be high. 

Ascorbate peroxidase (APX) is located in chloroplasts as well as cytosol and represents a key enzyme in the ascorbate cycle where it exchanges H_2_O_2_ by negotiation of ascorbate as an electron donor and formation of H_2_O [63]. In this study, APX activity increased under treatment with Pb depending on the concentration, which indicates its role in the defense mechanism under stress. Regarding GPX, its activity increased under Pb stress in the present investigation, and this is in harmony with the results of [64], where GPX is involved in removing ROS production under stress.

The increment in the SOD activity in the present work under lead stress is probably due to the reduction of superoxide radicals [65]. Superoxide radicals are considered to be the first radicals produced under stress, and SOD converts superoxide radicals quickly into H_2_O_2_ and O_2_. Thus, SOD plays an important part in scavenging free radicals [66].

Glutathione reductase (GR) is involved in the AsA-GSH cycle and performs a critical function in scavenging ROS under stress and maintains both AsA and GSH levels in living cells [67].

Various researchers suggested that ALA can alleviate stress through improving antioxidant enzyme activities while scavenging ROS [62,68,69] and this is what the results in the present investigation proved either in hydroponic or pot experiments. The activities of the measured enzymes in plants originated from water-primed seeds were higher than those primed in ALA and then treated with Pb and this might be due to the use of higher Pb concentrations. 

Priming of *Salvia* seeds in 10 or 20 mg L^−1^ ALA expressed an ameliorative effect on Pb toxicity. ALA is a common precursor of tetrapyrroles as well as a crucial growth regulator in higher plants and has been reported to be effective in improving photosynthesis and alleviating the adverse effects of various abiotic stresses in higher plants such as salinity stress in cucumber seedlings [70]. It has been described that ALA protects seedlings of plants against stress by preventing lipid peroxidation, induction of antioxidant enzyme activities, regulating endogenous hormones in addition to nutrient accumulation [70]. Also, ALA improved the tolerance against salt stress in *Isatis indigotica* [71].

## 5. Conclusions

Pb markedly reduced germination and protein content but increased MDA, H_2_O_2_ levels, and the antioxidant enzyme activities in *S. officinalis* (Sage) leaves. Priming seeds in ALA (10 and 20 mg L^−1^) decreased the levels of both H_2_O_2_ and MDA. In addition, it lowered the induced GPX, APX, SOD, and GR under Pb treatment. Hence, priming seeds in ALA is valuable for the alleviation of Pb stress in *S. officinalis* and possibly in other plants. Nonetheless, more investigations are crucial to evaluate the genuine mechanism of ALA in alleviating the heavy metal (Pb) stress in plants. 

## Data Availability

Data are available from the authors upon request.
